# The transfer temperature from slow cooling to cryogenic storage is critical for optimal recovery of cryopreserved mammalian cells

**DOI:** 10.1371/journal.pone.0259571

**Published:** 2021-11-16

**Authors:** Peter Kilbride, Julie Meneghel, Fernanda Fonseca, John Morris

**Affiliations:** 1 Cytiva, Cambridge, United Kingdom; 2 INRAE, AgroParisTech, UMR SayFood, Université Paris-Saclay, Thiverval-Grignon, France; University of Maryland, UNITED STATES

## Abstract

Cryopreservation is a key step for the effective delivery of many cell therapies and for the maintenance of biological materials for research. The preservation process must be carefully controlled to ensure maximum, post-thaw recovery using cooling rates slow enough to allow time for cells to cryodehydrate sufficiently to avoid lethal intracellular ice. This study focuses on determining the temperature necessary at the end of controlled slow cooling before transfer to cryogenic storage which ensures optimal recovery of the processed cell samples. Using nucleated, mammalian cell lines derived from liver (HepG2), ovary (CHO) and bone tissue (MG63) this study has shown that cooling must be controlled to -40°C before transfer to long term storage to ensure optimal cell recovery. No further advantage was seen by controlling cooling to lower temperatures. These results are consistent with collected differential scanning calorimetry data, that indicated the cells underwent an intracellular, colloidal glass transition between -49 and -59°C (Tg’i) in the presence of the cryoprotective agent dimethyl sulfoxide (DMSO). The glass forms at the point of maximum cryodehydration and no further cellular dehydration is possible. At this point the risk of lethal intracellular ice forming on transfer to ultra-low temperature storage is eliminated. In practice it may not be necessary to continue slow cooling to below this temperature as optimal recovery at -40°C indicates that the cells have become sufficiently dehydrated to avoid further, significant damage when transferred into ultra-low temperature storage.

## Introduction

It is widely recognised that, to achieve optimal cell recovery function, nucleated mammalian cells require controlled rate cooling for the early part of the cryopreservation process. Subsequently, the choice of endpoint temperature (ET), when the sample is transferred to ultracold storage, must secure optimal viability and function. Inevitably, a suboptimal choice of ET will increase cell mortality.

The lethal injuries that are acquired during controlled rate cryopreservation are primarily dependant on intracellular ice formation (IIF) and solute toxicity [1, 2]. Freezing of the extracellular medium effectively removes water from the solution as ice crystals, leaving a residual, more concentrated and as yet unfrozen, extracellular solution. Ice formation continues as cooling progresses, with the residual, extracellular solution becoming increasingly concentrated. This creates an osmotic gradient between the suspending solution and the cells of the sample that drives cellular dehydration (cryodehydration). The soluble cytoplasmic cell content thus concentrates as cooling progresses which, fortuitously for cell survival, reduces the risk of lethal IIF. A cooling rate that is too rapid will not allow sufficient cryodehydration to avoid IIF. Control of the cooling rate can be used, therefore, to control cryodehydration to limit the risk of IIF during cryopreservation and contribute significantly to post-cryopreservation cell survival [1, 3]. Prolonged exposure to high solute concentration in both the extra- and intracellular environments, will also impose toxic stresses on the cells. This is often seen when cooling proceeds too slowly. Consequently, the optimal cooling rate will be slow enough for cells to dehydrate sufficiently to avoid IIF yet rapid enough to limit any damaging, toxic effects to an acceptable level [2, 4].

The success of controlled cooling for cryopreservation has been widely reported for nucleated, mammalian somatic cells, where relatively slow rates are employed [2–8]. For example, immortalised T lymphocytes [4], porcine corneal endothelial cells [9] and liver spheroids cooled [10] at 0.1, 0.2 and 0.3°C min^-1^ respectively show reproducibly high post-thaw recovery, indicating that these rates allow for sufficient cryodehydration to minimise IIF and that toxicity in response to concentrating, extracellular and intracellular solutions was below critical levels. More rapid cooling rates e.g. 10°C min^-1^ will not allow typical, nucleated mammalian cells to cryo-dehydrate sufficiently to avoid IIF [4]. However, for optimum post-thaw survival of cells with a relatively high membrane permeability, such as red blood cells and sperm cells cooling at 10°C min^-1^ or faster (depending on species), is required [11–17]. For such cell types, it is only at these faster rates that the critical combination of cryodehydration and toxicity avoidance necessary for cell survival is achieved. However, evidence to support the choice of ET in nucleated non-reproductive cells before transfer to the selected storage temperature is sparse. For many systems including mammalian oocytes and embryos [18–23], ovarian tissue or embryonic cells ET values from -15 to -65°C have been employed [17, 18, 24–26]. Controlled cooling to temperatures as extreme as -80 to -120°C has also been reported [6, 14]. However, the differences in cellular structure, cryoprotective agent (CPA) used, optimal cooling rates, and cell membrane properties necessities study of this ET in non-reproductive nucleated cells.

When a sample is transferred from controlled rate freezing to an ultra-low storage temperature the cells must be sufficiently dehydrated to provide maximal protection against IIF, with a minimal influence of toxic factors. As free water leaves the cells during cryodehydration they deform and shrink, bringing intracellular structures, and proteins, into an increasingly close network with increasing viscosity. At ultra-low temperatures, this network will form into a colloidal glass. Once the colloidal glass has formed, lethal IIF cannot occur. The temperature at which this intracellular glass transition (Tg’i) occurs defines the ET, at which a cell can be considered as maximally dehydrated for successful cryopreservation [3, 27]. After reaching the ET no additional benefit will be gained by continuing cooling before transfer to the final, cryogenic storage temperature. To ensure secure, long term cryopreservation the storage temperature must be maintained below the glass transition of the extracellular medium (Tg’e), which is lower than the intracellular Tg’i [3]. The ET can be measured using differential scanning calorimetry (DSC)[3, 27, 28]. A recent example has shown that immortalized T lymphocytes cryopreserved in 10% v/v DMSO reach a maximally dehydrated state at -47°C, the measured Tg’i that indicated the colloidal glass transition event [3]. Taking this as the ET for optimal cell survival, then controlled rate cooling can be ceased and the cells transferred into ultralow storage with minimal risk of IIF [3]. Identifying ET accurately will limit the loss of valuable cells and eliminate unnecessary controlled cooling, providing significant clinical and resource benefits for regenerative medicine and cellular therapies. It is a central element in the development of standard operating procedures (SOPs).

In this study cell lines derived from Chinese hamster ovary (CHO), human liver (HepG2) and human bone (MG63) tissues have been selected as representing nucleated somatic cells and the effects of ET on post-thaw viability and function following controlled, slow cooling investigated. DSC has been used to measure the onset temperature of the intracellular glass transition (Tg’i) [3, 27] and a safe ET for practical cryopreservation derived for these cell lines.

Further, DSC measurement has been used to gain an initial insight into the impact of permeating CPAs on the Tg’i, using red blood cells as a model system. Given the mechanism by which the intracellular colloidal glass forms, it would be expected that a high protein concentration in slowly cooled cells would result in a high intracellular Tg’i. The addition of a cell-membrane permeating CPA, such as DMSO, which provides an additional intracellular solute, would be expected to lower the Tg’i as it reduces the rate of dehydration a cell experiences as cooling progresses. The impact of the permeating CPA DMSO on the colloidal glass transition of the porcine red blood cells relative to a medium without a permeating CPA was established.

## Methods

### Cell line culture

CHO (Chinese hamster ovary), HepG2 (Human liver) and MG63 (Human bone) cells were acquired from Sigma-Aldrich, Gillingham, UK (#85050302, # 85011430 and #86051601, resp.). CHO cells were cultured in Ham’s F12 medium (HyClone, Logan, UT, USA #SH30026.01), HepG2 cells in RPMI-1640 medium and MG63 cells in DMEM medium (Sigma-Aldrich #R8758 and #D5030 resp.). Basal media were supplemented with 10% v/v iron-fortified foetal calf serum (Sigma-Aldrich, #C8056), 2% v/v penicillin-streptomycin (Sigma-Aldrich, #P4333) and 1% v/v amphotericin B solution (Sigma-Aldrich, #2942). The MG63 medium was additionally supplemented with 20mM glutamine (Sigma-Aldrich # G3126). A supplemented medium is hereafter referred to as a complete culture medium (CCM). A series of T175 culture flasks (ThermoFisher Scientific, Roskilde, Denmark #10246131) containing 50 mL of the appropriate CCM were each seeded with 10^6^ of the selected cells and incubated in a humidified incubator (5% CO_2_ atmosphere) at 37°C. The CCM was changed on days 2, 4 and 7 post-seeding and cells harvested when reaching ≥ 70% confluency. The cells were trypsinised for a maximum of 15 minutes at 37°C using 10mL of pre-warmed 1X trypsin (Sigma-Aldrich, #59427C) in PBS per flask, stopped by the addition of the same volume of pre-warmed CCM. The sample was then transferred to 50 mL centrifuge tubes (Corning, from Sigma-Aldrich, #CLS430791) and centrifuged at 1200 rpm for 4 minutes in an Heraeus Megafuge 16 centrifuge equipped with a 3655-swinging bucket rotor (ThermoFisher Scientific). The supernatants were discarded, cell pellets pooled and then resuspended in a small volume of CCM. A 100 μL sample of the final cell suspension was removed for viability measurement at a density of 10^6^ cells mL^-1^.

### Cryopreservation

**Fig 1:**
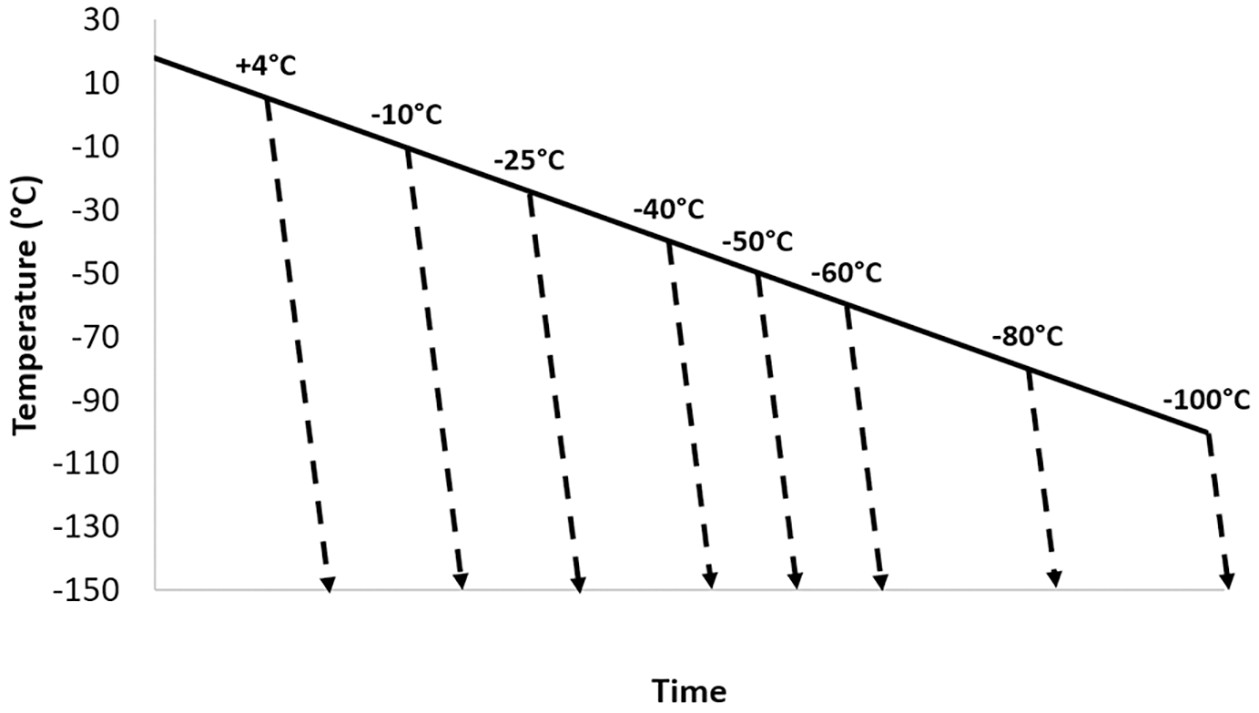
The experimental cooling profiles used for cryopreservation of HepG2, CHO and MG63 cells. 1ml-filled cryovials were cooled in a controlled rate freezer at 1°C min^-1^. At selected temperatures between +4° and -100°C samples were plunged directly into liquid nitrogen (dashed lines), where they cooled at a rapid. uncontrolled rapid rate. A second experiment with endpoint temperatures between -38 and -50°C was also carried out.

A cell suspension at 1.11x10^6^ cells/mL was prepared in CCM at room temperature, and DMSO (Sigma-Aldrich, #D4540) was added in a single-step to provide a final concentration of 10^6^ cells mL^-1^ in 10% v/v DMSO. Thereafter, 1mL aliquots were added to 2mL-cryovials (Corning, from ThermoFisher Scientific, #13429798) which were cooled to 4°C. These vials were then cooled at 1°C min^-1^ to the required ETs (Fig 1) in a VIA Freeze controlled rate freezer (Cytiva, Cambridge, UK). Five vials were removed at selected temperatures between 4°C and -100°C (± 2°C) and plunged directly into liquid nitrogen (LN_2_). Thermocouples were added to vials (n = 8) containing only the CPA mix to determine temperature profiles when plunging into LN_2_. Vials were stored below -140°C for at least 24 h before thawing (Fig 1). Vials were thawed in an automated device (SC2 VIA Thaw for vials, Cytiva, Cambridge, UK) taking c. 3 minutes. Thawing was confirmed visually by the absence of ice crystals before the contents were mixed and 25 μL of cell suspension was placed into each well of two columns of a 96-well plate (ThermoFisher Scientific, #10212811). Subsequently, 200 μL of CCM was added to each well to provide c.25,000 cells/well and a DMSO concentration below 1% v/v. Five vials were included on each plate, with edge columns containing cell-free culture medium to minimise edge effects. The plates were cultured in a humidified incubator at 37°C (5% CO_2_ atmosphere) and assessed at 24, 48 and 72 h post-thaw.

### Post-thaw cell assessment

Counts of adherent cells were made using the permeating nuclear stain Hoechst 33342 (NucBlue^™^ Live ReadyProbes^™^, ThermoFischer, #R37605) which emits blue fluorescence when bound to DNA. For each well to be assessed on the multi-well plate, 1 drop of NucBlue was added and the plate incubated at 37°C for 15 minutes as per manufacturer’s instructions. Fluorescent adherent cells were counted using a Cytell Imaging System at 10x magnification, 390nm_ex_/430nm_em_, with 20 individual images per sample at each timepoint (Cytiva, Preston, UK). Data were normalized to 1 at the -50°C ET, 24h post-thaw in order to compare differences directly between different cell types and timepoints.

Redox activity, indicating a level of active respiratory metabolism, was measured using Resazurin sodium salt (Sigma-Aldrich, #7017). The assay demonstrates the ability of cells to reduce non-fluorescent resazurin to fluorescent resorufin. Resazurin sodium salt was diluted in CCM to a final concentration of 0.005% w/v and 100 μL added to each well under study. Unless otherwise stated, a prolonged incubation time of 4 hours was chosen to eliminate background activity from dying cells. The multi-well plate was incubated for 4 hours at 37°C in a humidified incubator (5% CO_2_ atmosphere) and the total fluorescence emitted was measured in a microplate reader (Tecan, Männedorf, Switzerland) at 560nm_ex_/612nm_em_ using Magellan software (version 7.2, Tecan). Wells containing culture medium and Resazurin sodium salt but without cells provided background values to be subtracted from the experimental measurements. Data were normalized to 1 at the -50°C ET, 24 h post-thaw.

### Preparation of cells for differential scanning calorimetry

#### CHO, HepG2 and MG63 cells in 10% v/v DMSO

Cell pellets were used for DSC to detect intracellular glass transitions (Tg’i) as these are, typically, too weak to be detected in a more diluted state. Approximately 35x10^6^ cells in CCM containing 10% DMSO (v/v) were pelleted at 13,000 g for 10 minutes, then transferred to a 2-mL Eppendorf tube and pelleted again twice at 16,000 g for 5 minutes. The supernatant was discarded between each cycle to remove as much liquid as possible. Approximately 25 mg of the cell pellet was placed in each 50 μL Perkin Elmer DSC aluminium pans (#B0143017 and B0143003, Perkin Elmer, Villebon-sur-Yvette, France), and the pan covered and sealed (Universal Crimper Press, B0139005, Perkin Elmer) for DSC analysis.

#### RBCs (in saline)

Fresh, food grade porcine blood was acquired from a local abattoir, stored at 4°C in 12% v/v of citrate-phosphate-dextrose buffer as per Greening et al. 2010 [29] to prevent coagulation and used within 5 days. Samples were centrifuged at 13,000 g for 5 minutes and the supernatant containing plasma and white cell populations removed and discarded. The cells were resuspended in a saline (0.9% w/w) or DMSO (10% v/v) solution and rested for 5 minutes to allow equilibration of the intracellular and extracellular media. Samples were then centrifuged twice at 13,000 g for 5 minutes, with the supernatant removed after each centrifugation. Approximately 25 mg of the cell mass was placed in 50 μL Perkin Elmer DSC aluminium pans as for CHO, MG63, and HepG2 cells.

### Differential scanning calorimetry measurements

Measurements were made as described previously [3, 27, 30], on cell pellets and solutions, using a power compensation calorimeter (Diamond, Perkin Elmer LLC, Norwalk, CT, USA) equipped with a liquid nitrogen cooling accessory (CryoFill, Perkin Elmer). Temperature calibration was performed using cyclohexane (crystal-crystal transition -87.1°C) and mercury (melting point -38.6°C). For each measurement an empty pan was used as a reference. Linear cooling rates of 1, 2, or 10°C min^-1^ were applied between 20°C and -150°C and samples then warmed to > 0°C at 10°C min^-1^.

**Fig 2:**
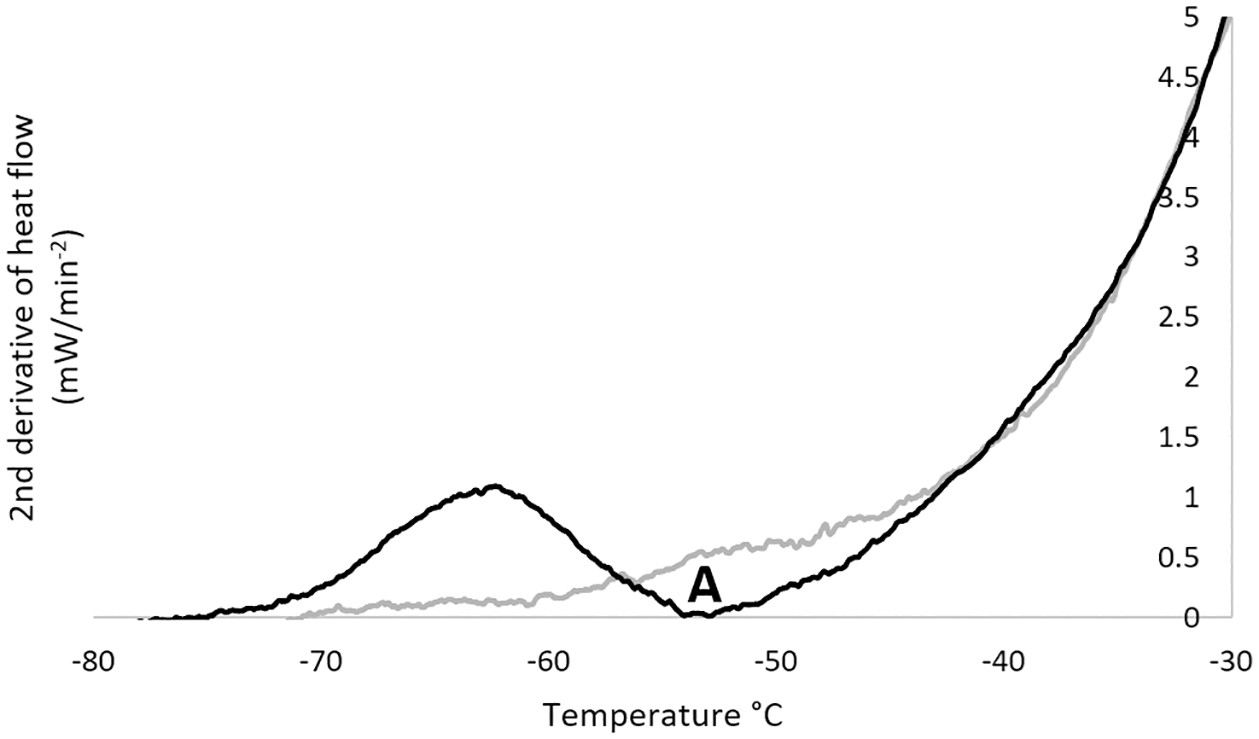
An example of a DSC trace, to which a second time derivative has been applied, used to measure Tg’i for a sample of HepG2 cells). A trace from a cell suspension is shown in black, together one for the cryoprotectant solution without cells (grey). For the cell suspension a transition is seen at point A (black) corresponding to the intracellular glass transition Tg’i.

To determine the glass transition temperature (Tg’, °C) heat flow data was recorded during warming [3, 27, 28]. Heat flow derivatives versus temperature were calculated to better visualise the transitions. Because the end of the glass transition event frequently overlapped with ice melting, generating a shoulder in the derivative of the heat flow, the second derivative was calculated to identify the peak value, an example trace is shown in Fig 2. Measurements were taken of the CPA solution in the absence of cells to identify cell-specific transitions. Intracellular transitions are gradual events and so Tg’i was identified as the peak (indicating the most rapid) change in heat flow 2^nd^ derivative (Fig 2). Glass transition events were characterised by the endset value of the peak of the second derivative of heat flow considered as the temperature of initial loss of molecular mobility during cooling, as described previously [3]. Results for each sample were obtained from at least three separate cooling-warming cycles. The extracellular glass transition temperature (Tg’e) occurs at a known value for DMSO solutions and was identified during DSC, allowing it to be excluded as a candidate for Tg’i [3, 28].

### Statistical analysis

For each ET considered, n = 5 independent biological samples (not replicates of the same sample) were produced and analysed. Differences among their means were analysed by one-way analysis of variance (ANOVA). When ANOVA showed significant differences, pairwise comparisons between means were subjected to Tukey’s post-hoc test. ANOVA and Tukey’s post-hoc test were performed in R (version 3.4.2 [31]) using R Commander 2.4–1 at a 95% confidence level.

## Results

### Post-thaw cell assessment/outcome

**Fig 3:**
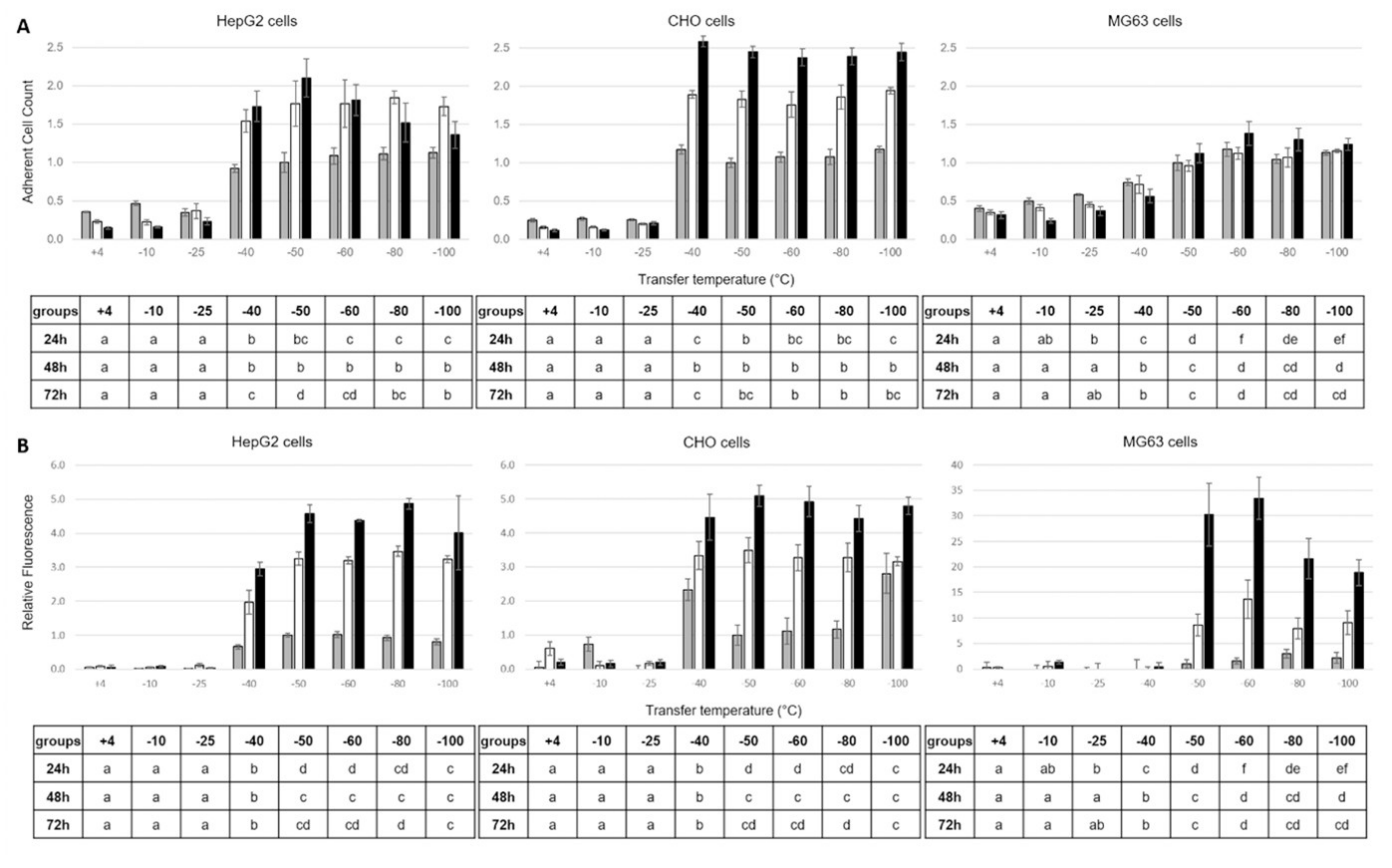
Adherent cell count (A), and redox activity (B) of HepG2 (left), CHO (center) and MG63 cells (right) measured after 24h (grey), 48h (white) and 72h (black) of culture post-thaw following cryopreservation at a controlled rate of 1°C min^-1^ down to endpoint temperatures (ET) between +4° and -100°C before plunging into liquid nitrogen. Grouping of ET for each analysis time point post-thaw is indicated by letters in the underneath tables, with distinct groups corresponding to P values < 0.05 and overlapping groups to 0.05 < P values < 0.10.

**Fig 4:**
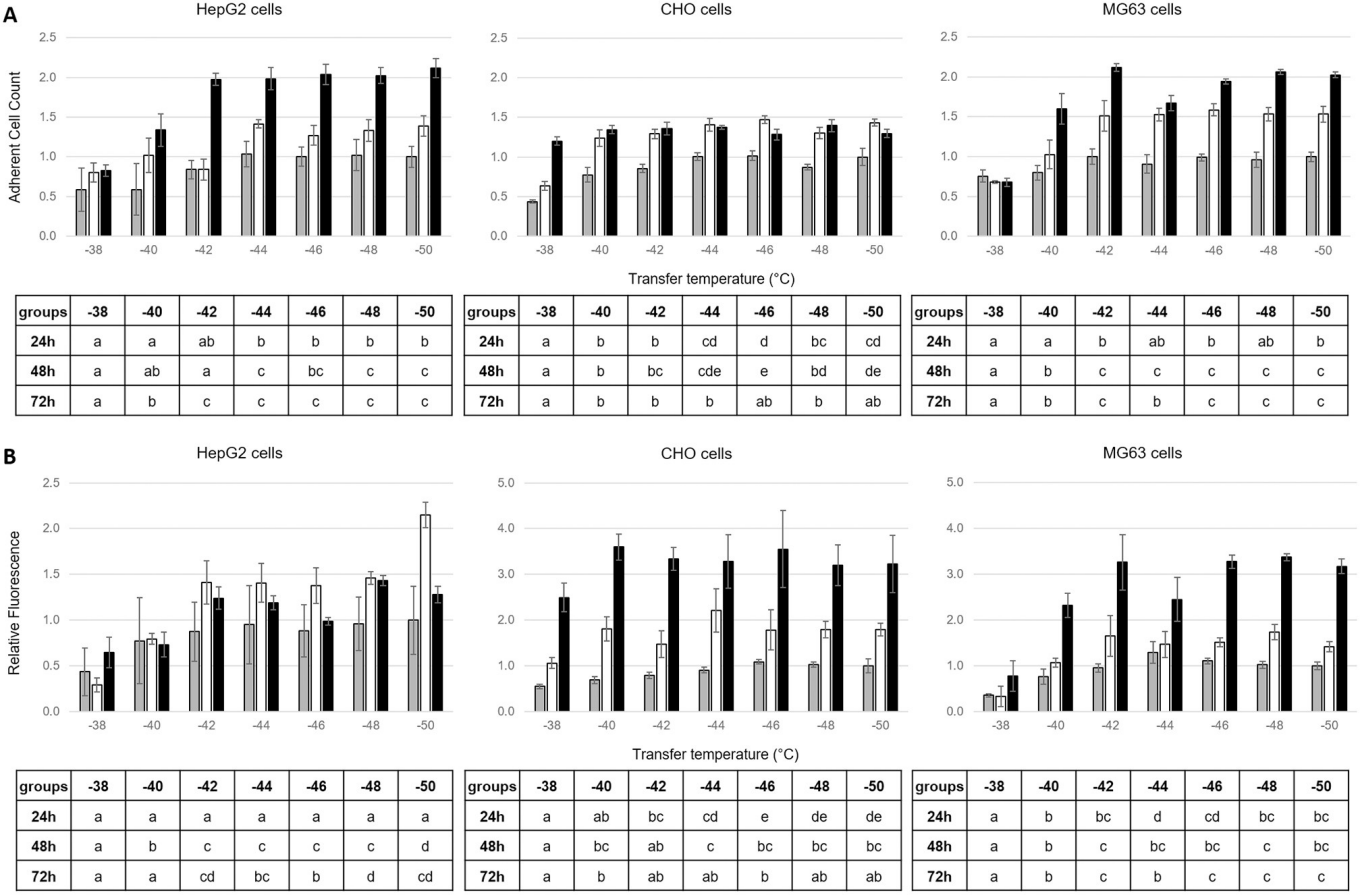
Adherent cell count (A), and redox activity (B) of HepG2 (left), CHO (centre) and MG63 cells (right) measured after 24h (grey), 48h (white) and 72h (black) of culture post-thaw following cryopreservation at a controlled rate of 1°C min^-1^ to endpoints at 2-degree intervals between -38° and -50°C before plunging into liquid nitrogen. Grouping of ET for each analysis time point post-thaw is indicated by letters in the underneath tables, with distinct groups corresponding to P values < 0.05 and overlapping groups to 0.05 < P values < 0.10.

Figs 3 and 4 present mean adherent cell numbers (Figs 3A and 4A) and redox activity (Figs 3B and 4B) measured after 24, 48 and 72h of culture post-thaw. This followed controlled cooling to a range of ETs between +4°C and -100°C (Fig 3) and smaller 2-degree intervals between -38°C and -50°C (Fig 4). Data are presented as histograms for HepG2 (left), CHO (middle) and MG63 cells (right). For each cell type and each measurement time point, the overall differences among the different ETs for adherent cell numbers and redox activity were always significant (*P* < 0.05). Tukey’s post-hoc tests were therefore conducted and the resulting statistical groupings between ETs are presented in the tables underneath each histograms, with groups statistically different from each other being assigned a different letter.

Transfer of cooling samples of HepG2 or CHO cells to LN_2_ before -40°C is reached is significantly detrimental to proliferation of recovered samples (*P* < 0.05) when compared to transfer at -40°C, after 24, 48 and 72 h of culture (Fig 3A). Transfer at -50°C or lower generates an equivalent level of recovery after 24 h of culture for both these cell lines (*P* > 0.10). The recorded lower proliferation activity of HepG2 cells between 48 and 72h of culture post-thaw for ETs ≤ -40°C is a consequence of rapid proliferation such that the cell cultures reached confluence. These data indicate that there may be a limited advantage in terms of cell proliferation in delaying the transfer beyond -40°C for CHO and HepG2 cells. The patterns of adherent cell counts are essentially reflected in the post-thaw redox activity for the HepG2 and CHO cell lines (Fig 3B), with the difference that no further statistically significant advantage is conferred in delaying the transfer beyond -50°C. The MG63 cell line shows consistently, and significantly, improved proliferation and redox activity (*P* < 0.05) with an ET at -50°C (Fig 3A and 3B).

To evaluate more precisely the necessary ET the post-thawed cell assessment also included 2°C intervals between -38°C and -50°C (Fig 4). It showed that post-thaw adherent cell numbers and redox activity were optimised for HepG2 cells when transfer was made after the samples reached at least -42°C, and -40°C for CHO and MG63 cells. Below these temperatures any further improvement appeared limited (Fig 4).

Upon transfer to LN_2_, samples took 6.7 ± 1.2 seconds to fall 20°C from the transfer temperature. The cooling rate reached a maximum of -412 ± 33°C min^-1^ 3.8 ± 1.1 seconds after being plunged into LN_2_.

### Differential scanning calorimetry measurements

The three nucleated cell lines cryopreserved with 10% v/v DMSO as the added CPA, and red blood cells cooled in a medium also with 10% v/v DMSO, exhibited the onset of the colloidal glass transition between -48.6 ± 4.9°C and -59.1 ± 1.1°C, with full results given in Table 1. Red blood cells prepared in isotonic saline without CPA addition, recorded a transition onset at -15°C. Extracellular glass transitions were observed at approximately -120°C in cell suspensions containing DMSO. Glass transition do not occur in saline or peptone-water, rather a eutectic forms where sodium chloride precipitates and the water component fully crystalizes [32].

**Table 1:** Intracellular glass transition temperature for a range of cell types, determined by DSC analysis. The temperature providing optimal cell recovery following cryopreservation of the nucleate, mammalian cell lines is presented as the safe temperature for transfer to long term storage.

Cell Type	Cryoprotective agent used	Colloidal Glass Transition Temp ± SD (°C)	Safe Transfer* Temperature (°C)
CHO (ovarian)	10% DMSO	-48.6 ± 4.9	-40 ± 2
HepG2 (liver)	10% DMSO	-53.7 ± 2.4	-42 ± 2
MG63 (bone)	10% DMSO	-59.1 ± 1.1	-42 ± 2
Red Blood Cells	10% DMSO	-51.2 ± 2.0	/
Red Blood Cells	Saline	-15.3 ± 0.5	/

*indicated by growth and function assay.

## Discussion

This study found biologically optimal ET for CHO, HepG2, and MG63 cells between -40±2 and -42±2°C (Table 1). This is in line with our earlier work with T lymphocytes, cryopreserved with DMSO, where the optimal ET was identified as between -40 and -50°C [3]. Slow, controlled cooling to between -30°C and -40°C before a LN_2_ plunge step has been shown as optimal for the successful cryopreservation of human umbilical vein endothelial cells [33]. Further, rat neural cells cryopreserved in 10% DMSO had a much improved post-thaw outcome when the ET following slow, controlled cooling occurred at -48.2°C compared with -24.0°C [26]. Collectively, this body of evidence lends strong support to the use of an ET of c.-40°C as appropriate for the safe transfer of slowly cooled somatic mammalian cell samples to cryogenic, long-term storage.

The intracellular colloidal glass transition for the nucleated, mammalian cells investigated, as measured by DSC (Table 1), occurs c.10 to 16°C below the optimal biological ET for transfer to cryogenic storage as determined by growth and function assay (Fig 3). This might indicate that the cells are sufficiently, if not maximally, dehydrated at slightly above the Tg’i. Given that the cells at -40°C are certainly close to maximally dehydrated, their cytoplasmic content will be extremely viscous [34] with little residual free water available to form ice, and so a safe a transfer to long term storage slightly above Tg’i as found in this study would be expected.

By the biologically determined ET, any possible molecular diffusion rates are extremely slow [34], effectively eliminating any opportunity for IIF before the intracellular colloidal glass transition is reached. The difference in biological ET and the determined Tg’I will be cell type dependent, with factors such as cells intracellular solute and vesicle composition, as well as tolerance to small amounts of IIF.

It should be noted that, while Tg’i is by convention defined as a single point, in reality this transition takes place over a range of temperatures, and this can be observed in Fig 2. Different cells, even in an approximately homogenous cell suspension will experience slightly different Tg’i based on their individual size and contents (which can be influenced by events such as cell cycle stage which would not typically synchronise between all cells in a population). This explains biologically why some cells survive transfer at temperatures as high as -38°C, even though the population as a whole has lower viability post-thaw. The lower average temperature for Tg’i compared with biological recovery suggests that cells are protected from rapid rates of cooling towards the start of the Tg’i process, likely due to the cells already dehydrated state and high viscosities present at the onset of the transition.

Time is also required for IIF to form on cooling. When plunging 1ml samples into LN_2_ at -40°C, the biologically acceptable temperature, samples will take only a few seconds to fall below Tg’i, which may occur before the maximum cooling rates following an LN_2_ plunge are reached (3.8 seconds from plunge to maximum cooling rates). This may not be long enough for IIF to form sufficiently (considering the viscosity at these low temperatures) to damage cells.

Consequently, the safe transfer of the slowly cooled samples to a sub-Tg’e storage temperature can take place at, or slightly above, the intracellular glass transition. Changing the concentration or type of CPA employed will affect ET and further work is needed to describe and understand the nature of the changes to be expected. However, at a practical level there is little significant benefit in delaying the transfer into storage beyond the ET determined from the data presented in Figs 3 and 4, unless there is a risk of transient warming on sample transfer.

The storage temperature must be maintained below the glass transition of the extracellular medium (Tg’e) which, in the presence of DMSO as a CPA, has been measured at between -120 and -123°C [3, 4, 27, 35]. Even though the cell interior becomes a colloidal solid at Tg’i, preventing metabolic biological activity and ice formation, maintenance below Tg’e is essential for secure and stable storage. In channels within the colloidal glass and in the extracellular freeze-concentrated medium in which the cells are entrapped, some small molecules will still have a level of molecular mobility. This has the potential to cause chemical damage to the cells [3, 36–38]. However, below Tg’e all such molecular activity effectively ceases, thereby eliminating molecular mobility issues [3, 8, 17, 27, 35].

The data presented in Table 1 shows a relatively high Tg’i for porcine red blood cells, in the absence of DMSO, that can be attributed to their relatively high protein content. The addition of DMSO as a CPA depressed the Tg’i for these red cells significantly, from -15.3±0.5°C to -51.2±2.0°C. This illustrates the effect a CPA of this type will have on the ET required for successful cryopreservation. A major mechanism of cellular protection provided by DMSO, after extracellular ice initiation, is a reduction in the toxicity of salts in the cryo-concentrated, unfrozen channels between ice crystals. This continues during cooling and provides a non-aqueous component for solutes to dissolve into [39]. Further, as a membrane-permeating protectant, DMSO will reduce the extent of dehydration a cell experiences by providing additional intracellular solute. This addition extends the time needed for adequate cryodehydration in the presence of DMSO (indicated by a lowered Tg’i), and is a factor in explaining why rapid cooling with DMSO during cryopreservation is usually harmful [4]. Such a protectant will also modify the physico-chemical properties of the intra- and extra-cellular compartments, modulating their viscosity [34]. Increased viscosity will slow molecular diffusion and consequently slow the cryodehydration of the cells, other physical events, such as membrane phase transitions and ice nucleation, which usually occur at or close to 0°C. Non-permeating CPAs, such as glycerol, have relatively high viscosity and so will also slow cryodehydration, with the inevitable effect of lowering the observed colloidal glass transition temperature [27, 34, 40].

## Conclusions

Controlled cooling from the first formation of ice within the system until close to the intracellular glass transition temperature is essential for the optimal recovery of cryopreserved cells. This intracellular glass transition occurs between -49°C and -59°C for HepG2, MG63, and CHO cells, with safe biological transfer temperatures of between -40°C and -42°C determined following growth and function assay of recovered cells. Practically when cryopreserving, maintaining controlled rate cooling somewhat below the safe transfer temperature may be preferable to minimise the risk of damage during any incidental, transient warming occurring when samples are transferred from the controlled rate freezer to long term storage.

## Supporting information

S1 Data (XLSX)

S2 Data (XLSX)
